# COVID-19 and emergencies in patients with diabetes: two case reports

**DOI:** 10.1186/s13256-020-02659-4

**Published:** 2021-02-02

**Authors:** Rajib Kumar Dey, Abdullah Isneen Hilmy, Hisham Ahmed Imad, Abdul Azeez Yoosuf, Ali Abdulla Latheef

**Affiliations:** 1 Department of Internal Medicine, Indira Gandhi Memorial Hospital, Male’, Maldives; 2 Mahidol-Osaka Center for Infectious Diseases, Faculty of Tropical Medicine, Mahidol University, Bangkok, Thailand; 3 Department of Viral Infections, Research Institute for Microbial Diseases, Osaka University, Osaka, Japan; 4 Technical Advisory Group, Health Emergency Operation Center, Male’, Maldives

**Keywords:** COVID-19, Diabetes, Diabetic ketoacidosis, Hyperosmolar hyperglycemic state, Diabetic emergencies, Pandemic

## Abstract

**Background:**

Maldives reported its first Coronavirus disease 2019 (COVID-19) case on March 7th, 2020. Since then more than 9400 positive cases and 33 deaths have been reported. Recently studies have shown that COVID-19 patients with diabetes had a poor prognosis and a higher mortality rate when compared to the non-diabetic patients. Poorly controlled diabetic patients had a higher incidence of complications like diabetic ketoacidosis (DKA) and hyperosmolar hyperglycemic state (HHS) which might have been precipitated by COVID-19. DKA and HHS are potentially lethal but preventable conditions. During this pandemic, although cases of uncontrolled diabetes are frequently reported, there is scarcity in reporting of cases with diabetic emergencies.

**Case presentation:**

Case 1 was a 53-year old Asian male, admitted on Day 10th of illness with DKA with acute kidney injury, and Moderate COVID-19. Case 2 was a 72-year old Asian male, admitted with mild COVID-19 who developed HHS with acute kidney injury on day 9 of illness. Both patients were managed conservatively in intensive care unit, with intravenous fluids and insulin.

**Conclusion:**

Clinicians should focus on close monitoring of diabetic patients with COVID-19, to prevent diabetic emergencies like DKA and HHS. It is important to aggressively manage these conditions for a favorable outcome.

## Introduction

The severe acute respiratory syndrome coronavirus 2 (SARS-CoV-2) outbreak began in China in December 2019 [1]. The World Health Organization (WHO) declared COVID-19 as a pandemic on March 11th, 2020 [2] and since then there have been over thirty million confirmed cases and over nine hundred thousand deaths worldwide [3]. Maldives confirmed its first case of COVID-19 on March 7th, 2020 and till date, more than nine thousand four hundred cases and thirty-three deaths have been reported [4].

Studies have shown that elderly age and people of any age with certain medical conditions like diabetes mellitus, cardiovascular disease, cancer, obesity, chronic obstructive pulmonary disease, and chronic kidney disease are at increased risk of severe disease and mortality from COVID-19 [5].

The prevalence of diabetes in 1590 Chinese patients with COVID-19 was 8.2%. However, the prevalence of diabetes increased to 34.6% in patients with severe COVID-19 [6]. In a recent study done in New York City (USA) in patients with COVID-19, the prevalence of diabetes and obesity was higher in individuals admitted to hospital than those not admitted to hospital (34·7% vs 9·7% for diabetes and 39·5% vs 30·8% for obesity, respectively) [7]. In Italy, it was reported that 35.5% of patients who died from COVID-19 had diabetes, a prevalence greater than 3 times that of the general population [8].

A survey done in England (UK) showed that out of the 23 804 patients with COVID-19 who died in hospital, 32% had type 2 diabetes and 1.5% had type 1 diabetes, with 2.03 and 3.5 times the odds of dying compared with patients without type 2 diabetes and type 1 diabetes respectively [9]. The French CORONADO study revealed 3% had type 1 diabetes, 88·5% had type 2 diabetes, 5·4% had other type diabetes, and 3·1% were diagnosed at admission [10]. All these studies have shown that diabetic patients with COVID-19 had a worse prognosis and a higher mortality rate.

Diabetic emergencies like DKA and HHS are life-threatening but preventable. These complications can be precipitated by any infectious disease including COVID-19. Here we describe two cases of critical COVID-19 complicated by DKA and HHS managed in the Maldives.

## Case presentation

### Case 1

A 53-year-old Asian male patient presented to the emergency department with a history of fever for 10 days with fatigue, myalgia, ageusia, hyposmia, and one episode of vomiting. The patient also complained of polydipsia, polyuria, and nocturia for 1 week with generalized weakness and mild breathlessness with non-productive cough for 2 days. He had a history of Hypertension and Diabetes mellitus but was not on medications. He was managing his blood glucose by lifestyle modification with diet and exercise.

**Fig. 1:**
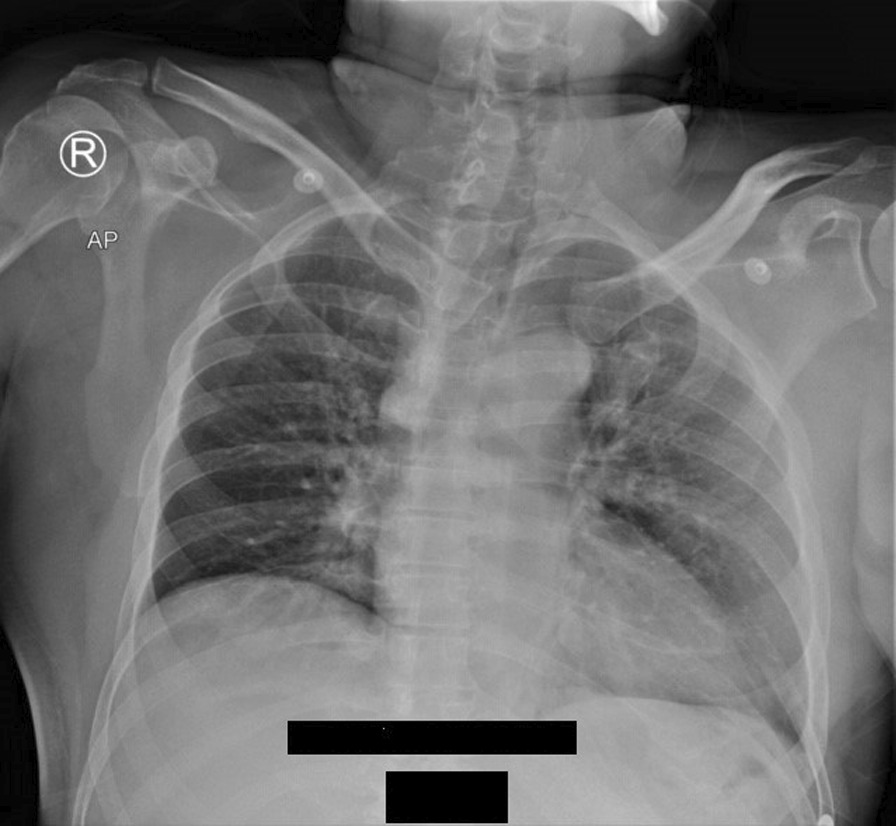
X-ray of case 1 showing bilateral airspace consolidations more prominent on the left side involving almost all zones

On initial evaluation, the patient was dehydrated with a heart rate of 108 beats per minute, blood pressure of 126/76 mmHg, temperature of 36.8 °C, respiratory rate of 24 breaths per minute, and oxygen saturation of 91% in room air. His blood investigations showed plasma blood glucose of 1543 mg/dl, HbA1c 13.0%, blood urea 130 mg/dl, serum creatinine 2.9 mg/dl, serum sodium 145 mEq/L, serum potassium 5.3 mEq/L, and urine analysis was positive for ketones (Table 1). His arterial blood gas analysis (ABG) revealed compensated metabolic acidosis with high anion gap. Chest x-ray revealed bilateral airspace consolidations, more prominent on the left side involving almost all zones (Fig. 1). The patient was planned for ICU admission and as a part of routine COVID-19 testing in our hospital, rRT-PCR was done which was positive. Hence, he was shifted to our make-shift COVID hospital and admitted to the ICU.

**Table 1:** Laboratory investigations in case 1

	25/07/2020	26/07/2020	27/07/2020	28/07/2020	29/07/2020	30/07/2020	02/08/2020
Hemoglobin, g/dl [range: 14–18]	14.7	12.8	13.4	12.0	12.3	12.3	12.7
Hematocrit, % [range: 40–54]	55.7	41.9	40.2	37	37.6	37.7	39
Leukocytes, 10^3^/ml [range: 5.0–10.0]	7.20	9.16	8.12	7.18	5.78	3.92	6.56
Neutrophils % [range: 45–74]	73.9	81.5	77.9	74.5	80.9	53.6	50.7
Lymphocytes, % [range: 16–45]	19.6	15.9	19.4	23.6	14.6	35.3	40.3
Platelets, 10^3^/ml [range: 150–450]	261	148	106	68.6	63.9	94.5	236
Urea, mg/dl [range: 19–44.1]	136.9	74.9	51.36	34.24	32.10	38.52	
Creatinine, mg/dl [range: 0.8–1.25]	2.9	1.4	1.19	0.90	0.78	0.83	
Na, mEq/L [range: 135–145]	149	164	159	149	139	139	
K, mEq/L [range: 3.5–5.1]	5.3	4.2	4.1	4.1	4.2	4.5	
AST, U/L [range: 05–34]	34	47	57	34	34	56	
ALT, U/L [range: 00–55]	19	28	33	30	28	43	
CRP, mg/ml [range: < 0.5]	17.6	19.8	9.71	14.10	17.90	14.29	4.03

HbA1c—13.0 %

Dengue Rapid: NS1 negative; Dengue IgM negative; Dengue IgG positive

SARS-CoV-2 rRT-PCR positive

Na: Sodium, K: Potassium, AST: Aspartate Transaminase, ALT: Alanine Transaminase, CRP: c-reactive protein, HbA1c: Glycosylated hemoglobin, NS1: nonstructural protein 1, SARS-CoV-2 rRT-PCR: severe acute respiratory syndrome Coronavirus 2 recombinant reverse transcriptase polymerase chain reaction

The patient was started on empirical antibiotics, oxygen supplementation through nasal prongs, and DKA management was initiated mainly with cautious administration of intravenous fluids, insulin therapy, and potassium correction. All other possible community-acquired pathogens were excluded.

The patient’s DKA and acute kidney injury (AKI) improved gradually which was evident by the normalization of renal function and serum sodium levels. He also developed thrombocytopenia during the course of treatment which improved over a week. The patient also showed improvement in his breathing as his oxygen saturation rose to 97% in room air and his repeat chest x-ray revealed resolution of consolidations. On the 7th day of admission, he was shifted out of ICU to the general ward in stable condition.

### Case 2

A 78-year-old Asian male was brought to our make-shift COVID-19 hospital after being tested positive for rRT-PCR. The patient had a history of diabetes mellitus, hypertension, and recurrent ischemic stroke. He was on regular medications which included antiplatelets, statins, losartan, and oral hypoglycemic agents. On admission, the patient had a mild fever and dry cough for 2 days, and his examination revealed a pulse rate of 70 beats per minute, blood pressure of 150/70 mmHg, temperature of 37 °C, SaO_2_ of 96% in room air, and respiratory rate of 20 per minute. His blood glucose was 90 mg/dl and HbA1c 6.6%. On the 9th day of admission, the patient gradually became drowsy and less responsive to general commands. On examination, his GCS was 10/15, pulse rate of 124 beats per minute, blood pressure of 180/100 mmHg, respiratory rate of 26 per minute, and oxygen saturation of 95% at room air. On chest auscultation, there was wheeze bilaterally present and crepitations on right infrascapular area.

**Fig. 2:**
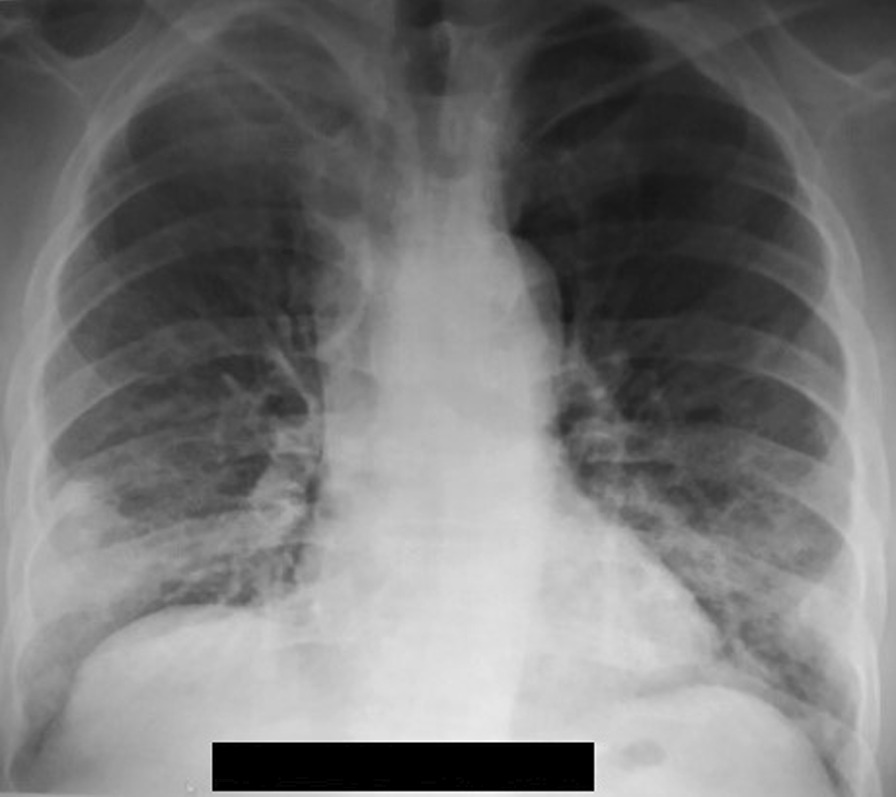
X-ray of case 2 showing bilateral ill-defined airspace consolidations involving mostly lower zones with peripheral distribution

His blood investigation showed a blood glucose level of 626 mg/dl, blood urea 64 mg/dl, serum creatinine 1.77 mg/dl, serum sodium 167 mEq/L, serum potassium 4.2 mEq/L, serum osmolality 378 mOsm/kg, urine analysis was negative for ketones (Table 2) and his arterial blood gas (ABG) analysis showed compensated metabolic acidosis. Chest x-ray revealed features of bilateral ill-defined airspace consolidations involving mostly lower zones with peripheral distribution (Fig. 2). Hence patient was shifted to ICU with the diagnosis of HHS with AKI and management was initiated with careful fluid administration, insulin therapy, and empirical antibiotics. All other possible co-infections were excluded. The patient gradually improved over a period of 1 week and was shifted out of ICU in stable condition.

**Table 2:** Laboratory investigations in case 2

Hemoglobin g/dl [range: 14–18]	11.3	11.4	10.5	9.1	11	9.2
Hematocrit, % [range: 40–54]	34	30	33	28	34.2	27
Leukocytes, 10^3^/ml [range: 5.0–10.0]	8.37	7.71	14.7	15.5	11.2	11.0
Neutrophils, % [range: 45–74]	70.1	63.4	91.7	93	82.2	80
Lymphocytes, % [range: 16–45]	16.0	21.9	3.9	4.7	13.2	11.1
Platelets, 10^3^/ml [range: 150–450]	174	180	240	179	180	176
Urea, mg/dl [range: 19–44.1]	59.9	44.9	64.2	59.9	29.96	
Creatinine, mg/dl [range: 0.8–1.25]	1.48	1.22	1.77	1.28	0.69	
Na, mEq/L [range: 135–145]	133	133	167	148	140	138
K, mEq/L [range: 3.5–5.1]	4.8	4.4	4.2	4.7	4.5	3.5
AST, U/l [range: 05–34]	23	26	22			
ALT, U/l [range: 00–55]	15	17	21			
CRP, mg/ml [range: < 0.5]	0.37	0.68	5.94	1.08	1.94	0.37

HbA1c—6.6 %

SARS-CoV-2 rRT-PCR positive

Na: Sodium, K: Potassium, AST: Aspartate Transaminase, ALT: Alanine Transaminase, CRP: c-reactive protein, HbA1c: Glycosylated hemoglobin, SARS-CoV-2 rRT-PCR: severe acute respiratory syndrome Coronavirus 2 recombinant reverse transcriptase polymerase chain reaction

## Discussion

We have described two cases of diabetic emergencies precipitated by COVID-19. The first case was admitted with DKA complicated by pneumonia who recovered well. The second case was admitted with HHS complicated by bilateral pneumonia who had a delayed recovery. To our knowledge, this is the first case report of COVID-19 with DKA, and HHS in the Maldives.

Older age and male sex have been associated with increased severity of COVID-19. In a study done in China, the overall case-fatality was 2.3% but it increased to 14.8% in individuals aged 80 years and older [11]. The prevalence of diabetes increases with age and hence the average age of COVID-19 patients with diabetes is older than the non-diabetic ones. Another study done on patients with COVID-19 with and without diabetes reported that the survivors were younger than the non-survivors and an age of 70 years and older was an independent risk factor for in-hospital death [12].

Severe acute respiratory syndrome coronavirus 2 (SARS-CoV-2) binds with angiotensin-converting enzyme 2 (ACE2) receptors which are highly expressed in pancreatic beta cells, lungs, kidneys, and small intestines [13]. ACE2 is the main enzyme in the renin-angiotensin-aldosterone system (RAAS) system that catalyzes the conversion of angiotensin II to angiotensin I. It is possible that the entry of SARS-CoV-2 into the pancreatic islet cells may cause direct injury to the beta cells leading to insulin deficiency [14]. The viral entry may also cause the downregulation of ACE2 enzyme which may lead to unopposed angiotensin II, which further impedes insulin secretion [15]. Angiotensin II stimulates aldosterone secretion which leads to increased risk of hypokalemia. Therefore, it be necessary for a higher potassium supplementation in order to continue intravenous insulin to suppress ketogenesis [15].

A high glucose concentration has been shown to be an independent predictor of death and morbidity in COVID-19 [16]. Diabetic emergencies occur due to insulin deficiency aggravated by hyperglycemia, dehydration and acidosis-producing derangements in intermediary metabolism. The most common causes are underlying infection, disruption of insulin treatment and new onset of diabetes. In both our cases the aggravating factor was noted to be COVID-19.

In case 1, the patient presented on the 10th day of illness when he was diagnosed with DKA with pneumonia and in case 2, the patient was initially admitted with mild symptoms of COVID-19 but developed HHS with bilateral pneumonia on the 9th day of illness. Both the patients were managed in the ICU. Conservative intravenous fluid was administered by using boluses instead of a continuous drip. After every bolus, clinical markers of perfusion like mean arterial pressure, urine output measurement, capillary refill time (CRT), heart rate and lactate levels were monitored. And as patients with COVID-19 are more prone to develop pulmonary edema [17], patient’s respiratory rate was monitored closely and chest auscultation was done at regular intervals in between fluid boluses [18].

Hyperglycemia in both patients were initially managed with Intravenous insulin infusion which was adjusted according to hourly blood glucose levels and was later changed to subcutaneous therapy. Serum electrolytes were closely monitored. Both patients had developed acute kidney injury along with the hyperglycemic crisis which gradually resolved within three days following fluid repletion. Empirical antibiotics were used in both the patients along with supplemental oxygenation via nasal prongs. Both the patients recovered over a period of 1 week.

## Conclusion

It is important for physicians to be vigilant in diabetic patients admitted with COVID-19 as they are at increased risk of developing complications. Diabetic emergencies like DKA and HHS can be precipitated by COVID-19 infection, and if not recognized early and treated promptly, it may lead to catastrophic outcomes. Clinicians should focus on close monitoring of diabetic patients with COVID-19, to prevent diabetic emergencies like diabetic ketoacidosis and hyperosmolar hyperglycemic state. As diabetes and COVID-19 are both highly prevalent in the Maldives, a high degree of suspicion is required to diagnose diabetic ketoacidosis and hyperosmolar hyperglycemic state timely for a better prognosis. In patients who develop diabetic emergencies, the intravenous fluid should be used judiciously with careful attention to markers of perfusion, to prevent volume overload and pulmonary edema.

## Data Availability

Not applicable.
